# Isolation and Characterization of a Duck Hepatitis A Virus Type 3 SH52 Strain in Ducklings

**DOI:** 10.3390/microorganisms13122652

**Published:** 2025-11-22

**Authors:** Minfan Huang, Dun Shuo, Yifei Xiong, Mei Tang, Yufei Wang, Xue Pan, Chunxiu Yuan, Qinfang Liu, Zhifei Zhang, Qiaoyang Teng, Bangfeng Xu, Xiaona Shi, Minghao Yan, Peirong Jiao, Zejun Li, Dawei Yan

**Affiliations:** 1 College of Veterinary Medicine, South China Agricultural University, Guangzhou 510642, China; 2 Department of Avian Infectious Diseases, Shanghai Veterinary Research Institute, Chinese Academy of Agricultural Sciences, Shanghai 200241, China; dun.shuo@hotmail.com (D.S.); nzhangzhifei@163.com (Z.Z.);; 3 College of Animal Sciences, Fujian Agriculture and Forestry University, Fuzhou 350002, China

**Keywords:** duck hepatitis A virus type 3, phylogenetic analysis, pathogenicity

## Abstract

Duck hepatitis A virus type 3 (DHAV-3), an avian-specific pathogen primarily impacting ducklings, poses a significant threat to the duck farming industry by causing high mortality rates. A DHAV-3 strain SH52 was isolated from the diseased ducks and phylogenetic analysis of the whole genome revealed that the DHAV-3 SH52 belongs to the prevalent strains in China. The DHAV-3 SH52 strain replicates at high levels in various organs of 8-day-old specific pathogen-free (SPF) shelducks, causing pathological damage and leading to high lethality in 8-day-olds following intramuscular infection. The findings of this study provide an ideal animal challenge model for evaluating the efficacy of DHAV-3 vaccines in the future.

## 1. Introduction

Duck hepatitis A virus (DHAV) is a single-stranded positive-stranded RNA virus belonging to the *Picornaviridae* family and *Avihepatovirus* genus [1,2]. Since its emergence in 1945, the serotype classification of duck viral hepatitis has undergone several changes due to the virus’s spread and mutation. In the Ninth Classification Report of the International Committee on Taxonomy of Viruses (ICTV) [3], the original DHV-1 [4], the Taiwanese duck hepatitis virus [5], and the Korean duck hepatitis virus [6] were reclassified as duck hepatitis A virus type 1 (DHAV-1), DHAV-2, and DHAV-3, respectively. Among these, DHAV-3 primarily affects ducklings under one week of age, characterized by a short incubation period, rapid onset, swift transmission, and a brief disease course. In artificial infections, clinical symptoms and death can occur as early as 24 h post-infection [7], with mortality rates reaching 90% to 100%. This makes DHAV-3 one of the most severe infectious diseases threatening the duck farming industry. Infected ducklings initially show signs of depression and loss of appetite, which rapidly progress to neurological symptoms such as generalized convulsions and opisthotonos. Common postmortem findings include enlargement and hemorrhage of the liver, spleen, and kidneys. In recent years, DHAV-3 has also been reported to cause non-typical hepatitis symptoms, such as pancreatitis, in ducklings [8]. In China, DHAV has an incidence rate of approximately 12% and a mortality rate of about 11%, with DHAV-3 being the predominant circulating strain, accounting for roughly 49% of reported cases [9]. Outbreaks of DHAV-3 are typically marked by acute onset, severe hepatic necrosis, and high mortality, resulting in significant economic losses for the duck breeding industry [10,11]. The virus can spread rapidly within flocks through direct contact, contaminated feed and water, and potentially via vertical transmission through eggs, posing serious challenges for disease management [12,13]. While there is no evidence that DHAV-3 can infect humans or cause human disease, thus posing no zoonotic risk, this virus poses a significant threat to animal health and food security. It has emerged as a major global concern for the duck farming industry, leading to substantial economic losses.

Previous studies have investigated the pathogenicity of different DHAV-3 strains [14,15]. And the results showed that DHAV-3 strains from different regions exhibit notable differences in molecular characteristics and pathogenicity. Molecular epidemiological analyses have further revealed that DHAV-3 can be subdivided into five genetic sub-clades (DHAV-3 a–e), reflecting substantial genetic heterogeneity among circulating strains. Whole-genome sequencing revealed that strains classified based on partial gene sequences were often misclassified, underscoring the critical role of whole-genome analysis in accurate classification [16,17]. Additionally, frequently detected recombination events—particularly concentrated in the 5′ untranslated region (UTR) and upstream of the capsid-encoding region—may have facilitated antigenic variation and immune escape [18]. This suggests that DHAV-3 continues to undergo genetic diversification, which may influence its virulence, host range, and vaccine efficacy [19]. Therefore, a thorough understanding of the genetic and biological characteristics of locally circulating strains is essential for elucidating their transmission dynamics, guiding prevention and control strategies, and advancing the development of effective vaccines [20]. Although molecular epidemiological studies on DHAV-3 have been relatively abundant both domestically and internationally, data on the genetic evolution and pathogenicity characteristics of strains in certain regions of China, particularly the East China region, remain limited.

In this study, a DHAV-3 strain SH52 was isolated and identified from a suspected case of duck hepatitis A in a duck farm in Jiangsu, China. The molecular characteristics were determined through whole-genome sequencing and phylogenetic analysis, and the pathogenicity and tissue distribution were evaluated through animal infection experiments.

## 2. Materials and Methods

### 2.1. Eggs, Animals, and Reagents

SPF shelduck eggs were purchased from the National Laboratory Animal Resources Bank of Poultry (Harbin, China) and conventional shelduck eggs were purchased from local duck farms (Nanzhuang Seedling and Poultry Cooperative of Jianhu County, Yancheng, China). Eight-day-old SPF shelducks were hatched and reared in the isolators. The viral DNA/RNA rapid extraction kit was purchased from Foshan Aowei Biotechnology Co., Ltd. (Shanghai, China). The DL2000 marker, Green Taq Mix, M-MLV(H-) Reverse Transcriptase, dNTP Mix (10 mM each), Random N9 Primers, and Murine RNase inhibitor were purchased from Nanjing Vazyme Biotech Co., Ltd. (Nanjing, China). The agarose gel DNA recovery kit (enhanced) was purchased from Tiangen Biochemical Technology (Beijing) Co., Ltd. (Beijing, China).

### 2.2. Viral (RT)-PCR Detection

Clinical tissue samples were collected from diseased ducklings, finely chopped, transferred into 2.0 mL tubes, and weighed. Sterile PBS (1.0 mL for each 1.0 g sample) and one sterile steel bead were added into the tubes, then the samples were homogenized in a tissue grinder. Following homogenization, the samples were centrifuged at 13,000× *g* for 10 min. The supernatant was collected, aliquoted, and stored at −80 °C. For DNA and RNA extraction, 200 μL of supernatant was processed with a viral DNA/RNA rapid extraction kit according to the manufacturer’s instructions. The extracted RNAs were then reverse-transcribed into cDNAs with Random N9 primer, under the following conditions: 30 °C for 10 min, 42 °C for 60 min, and 72 °C for 10 min.

This was performed to detect common duck viruses, including duck parvovirus (DPV), duck circovirus (DuCV), duck adenovirus serotype 3 (DAdV-3), duck hepatitis A virus type 3 (DHAV-3), novel duck reovirus (NDRV), duck Tembusu virus (DTMUV), duck egg-reducing syndrome virus (DERSV), and duck hepatitis A virus type 1 (DHAV-1). The DNAs and cDNAs were used as templates for PCR amplification (Supplemental Table S1) under the following conditions: 95 °C for 3 min, (95 °C for 15 s, 56 °C for 15 s, 72 °C for 40 s) × 30 cycles, and 72 °C for 10 min. PCR products were analyzed by 1.0% agarose gel electrophoresis.

### 2.3. Virus Isolation, Purification, and Titration

DHAV-3 PCR-positive samples were isolated and purified in SPF duck embryos by endpoint dilution method for three rounds. Briefly, 0.1 mL of the DHAV-3-positive sample was injected into the yolk sac of 9-day-old SPF duck embryos, with three replicates per group. Inoculated eggs were incubated at 37 °C for seven days and candled every 24 h to check if the embryo was alive. The allantoic fluid from dead duck embryos was collected and diluted for further purification. The allantoic fluid from dead duck embryos at the highest dilution was collected and the virus was titrated and designated as a DHAV-3 SH52 strain after three rounds of purification. The median embryo lethal dose (ELD_50_) was calculated using the Reed–Muench method.

### 2.4. Whole-Genome Sequencing and Analysis

To determine the full sequence of SH52, the RNAs was first reverse-transcribed to cDNAs using primer Oligo dT-RA (Supplemental Table S1). Based on the published DHAV-3 gene sequence (GenBank accession No. PQ777123.1), two pairs of specific primers (DHAV-3-P1-F/R and DHAV-3-P2P3-F/R) targeting the overlapped segments of the whole genome were designed using SnapGene DNA (Supplemental Table S1) and synthesized by Shanghai Tsingke Biotec Co., Ltd. (Shanghai, China). The RT-PCR products, covering the whole genome of the DHAV-3 SH52 strain, were purified and subjected to homologous recombination into the pCAGGS vector for sequencing. Sequence homology was assessed against other 37 full-length DHAV-3 genomes in GenBank, using DNAStar 7.1 for alignment and MEGA 12.0 for phylogenetic reconstruction. Evolutionary relationships based on the whole genomes were included in the phylogenetic analysis via the Neighbor-Joining (NJ) method with 1000 bootstrap replicates. The genotypic classification of DHAV-3 strains (sub-clades 3-a to 3-e) was conducted with reference to the established scheme based on full-length genome sequences [16,21,22].

### 2.5. Duck Experiments

To determine the pathogenicity and mortality of the DHAV-3 SH52 in ducklings, every ten 8-day-old SPF ducklings were randomly assigned into one of seven isolators. The ducks in different challenge groups were intramuscularly (i.m.) injected with 0.1 mL of DHAV-3 SH52 strain, containing 10^6.5^ ELD_50_, 10^5.5^ ELD_50_, 10^4.5^ ELD_50_, 10^3.5^ ELD_50_, 10^2.5^ ELD_50_, and 10^1.5^ ELD_50_ of the virus, respectively. Meanwhile, the ducks in the control group were i.m. injected with the same volume of sterile PBS. Daily clinical signs including water/food intake and death of the ducks were monitored in each group for seven days post-challenge. Samples of hearts, livers, spleens, lungs, and kidneys of three dead ducklings in the 10^6.5^ ELD_50_ challenge group were randomly collected and the viral titers were determined by using nine-day-old conventional duck embryos. For histopathological study, the heart, liver, spleen, lung, and kidney were fixed in 4% paraformaldehyde, sectioned, and stained with hematoxylin and eosin.

To systematically characterize the gross and histopathological changes induced by DHAV-3 SH52 and to better understand its pathogenicity in ducklings, a disease evaluation system for different tissues was introduced [14]. Briefly, this system employed a semi-quantitative scoring method adapted from previous studies, with the following criteria: “-” for no lesions, “+” for mild (5–25% involvement), “++” for moderate (26–50%), and “+++” for severe (>50%).

### 2.6. Statistical Analysis

Data derived from animal experiments were calculated as means ± standard deviation (SD).

## 3. Results

### 3.1. Viral RT-PCR Detection

**Figure 1:**
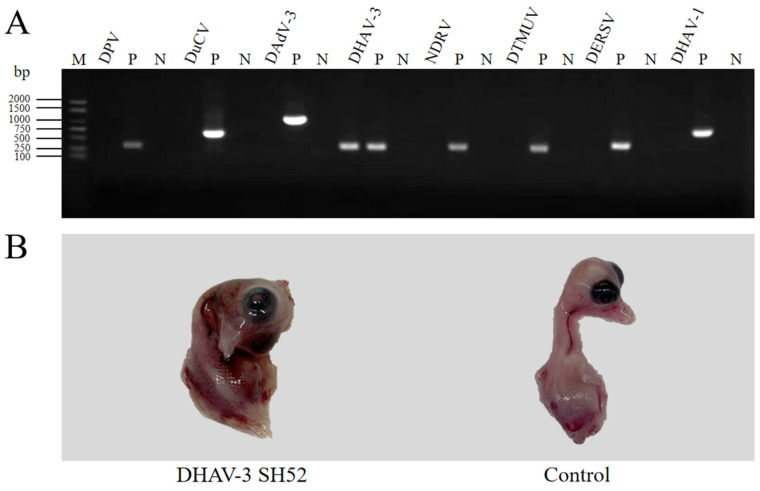
Identification and isolation of the DHAV-3 SH52 strain. (**A**) Tissue samples were tested by RT-PCR and PCR for common duck viruses. The RT-PCR and PCR analysis revealed 286 bp of the DHAV-3 target sequence in lane 11. Lanes 2 to 10 and lanes 14 to 25, which included DPV, DuCV, DAdV-3, NDRV, DTMUV, DERSV, DHAV-1, and their respective positive controls (P) and negative controls (N), extracted from the healthy ducklings, showed no interference with the target sequence. Lane 1 was served as the DL2000 marker (M). (**B**) DHAV-3-positive samples were inoculated into SPF duck embryos. The inoculated duck embryos died within 72 h post-inoculation, exhibiting systemic edema and hemorrhages, while the control duck embryos exhibited no gross pathological lesions.

Tissue samples from diseased ducklings were homogenized and centrifuged, and the total DNAs/RNAs of the supernatant were extracted. RNAs were further reverse-transcribed to cDNAs. To detect common viruses, including DPV, DuCV, DAdV-3, DHAV-3, NDRV, DTMUV, DERSV, and DHAV-1, DNAs and cDNAs of the samples were amplified by PCR based on specific primers, respectively. PCR products were electrophoresed on 1.0% agarose gels, revealing 286 bp of the DHAV-3 target sequence in lane 8, while yielding negative results for other tested viruses (Figure 1A).

### 3.2. Virus Isolation, Purification, and Titration

The virus replicated efficiently in SPF duck embryos in the eggs, inducing mortality within 24–120 h post-inoculation. The dead embryos demonstrated developmental retardation, generalized edema, and extensive petechiae in the cervical subcutaneous tissue, which differed from uninfected control embryos (Figure 1B). After three rounds of purification in SPF duck embryos using endpoint dilution method, the virus was named as DHAV-3 SH52 and the titer was determined to be 10^6.5^ ELD_50_/0.1 mL.

### 3.3. Whole-Genome Sequencing and Phylogenetic Analysis of the DHAV-3 SH52

To determine the whole genome of the SH52, two PCR products, covering the whole genome of DHAV-3 SH52, were amplified, purified, and homologously recombined into the pCAGGS vector for sequencing. The SH52 contained genomes of 7791 nucleotides in length, and posed a 6756-nucleotide single ORF, encoding 2251 amino acid polyprotein. The polyprotein appeared to be cleaved to produce three structural proteins (VP0, VP1, and VP3) and seven nonstructural proteins (2A, 2B, 2C, 3A, 3B, 3C, and 3D). In addition, the SH52 contained a 652-nucleotide 5′-UTR and a 383-nucleotide 3′-UTR. The full-length gene sequence of SH52 was submitted to Genbank under accession number PX380846. Sequence analysis revealed 91.7–98.9% nucleotide identity with published DHAV-3 genomes, demonstrating a high conservation.

**Figure 2:**
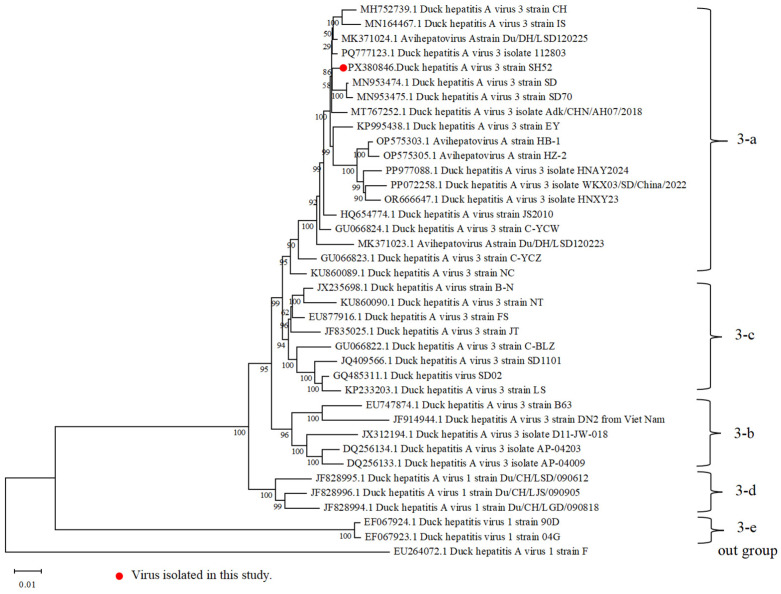
Phylogenetic analysis based on the whole gene of DHAV-3. Sequences were aligned by Clustal W method and phylogenetic trees were generated using the NJ method in MEGA 12.0 software. For each strain, the GenBank accession number, strain type, and name are displayed.

To determine the phylogenetic relationship of DHAV-3 SH52 and other reported DHAV-3 strains, the full length of SH52 was compared with those of representative DHAV-3 strains and a DHAV-1 strain, used as an outgroup. Phylogenetic analysis based on the complete genome revealed that DHAV-3 SH52 belongs to sub-clade 3-a, a distinct evolutionary clade of strains prevalent in China in recent years (Figure 2). It shared the closest phylogenetic relationship with strain DHAV-3 A/dk/CHN/AH07/2018 (GenBank accession No. PQ777123.1), exhibiting a nucleotide identity of 98.9%.

### 3.4. Pathogenicity in Ducklings

**Figure 3:**
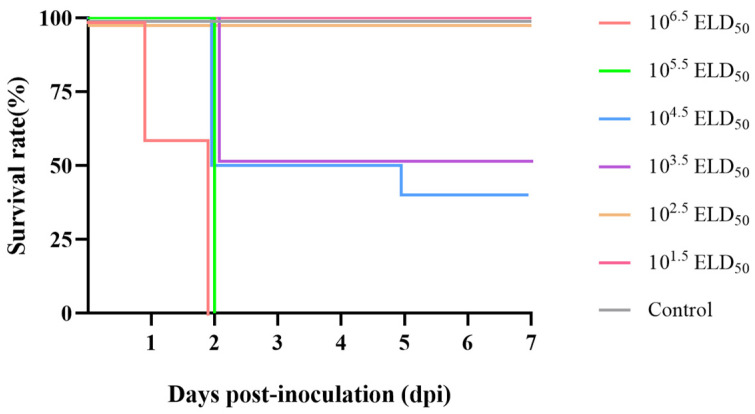
Survival of ducklings inoculated with different titers of DHAV-3 SH52. Ducklings i.m. inoculated with different titers of DHAV-3 SH52 exhibited varying survival rates. Survival rates in experimental groups inoculated with 10^6.5^ ELD_50_ to 10^1.5^ ELD_50_ were 0%, 0%, 40%, 50%, 100%, and 100%, respectively. No deaths occurred in the control group.

To evaluate the pathogenesis of DHAV-3 SH52 at different viral doses, every ten 8-day-old SPF shelducks were infected with 10^6.5^ ELD_50_, 10^5.5^ ELD_50_, 10^4.5^ ELD_50_, 10^3.5^ ELD_50_, 10^2.5^ ELD_50_, and 10^1.5^ ELD_50_ of the virus through the i.m. route, respectively. The six experimental groups of ducklings exhibited varying degrees of clinical symptoms after infection. Those in the groups inoculated with higher viral loads developed clinical signs such as lethargy, reduced appetite, ataxia, and paddling movements before dying within 3 days post-inoculation (dpis). Dead ducklings typically presented with the characteristic opisthotonic posture. During 7 days of observation, the survival rates of ducks in different groups were as follows: in the 10^6.5^ ELD_50_ and 10^5.5^ ELD_50_ groups, all ducklings died within 2 dpis; in the 10^4.5^ ELD_50_ group, five of ten ducklings died at 2 dpis, and one died at 5 dpis, achieving a 40% survival rate; in the 10^3.5^ ELD_50_ group, there were five deaths at 2 dpis, resulting in a 50% survival rate; in the 10^2.5^ ELD_50_ and 10^1.5^ ELD_50_ groups, no mortality were observed; in the control group, all ten ducks exhibited no clinical signs and deaths throughout the observation period (Figure 3).

**Figure 4:**
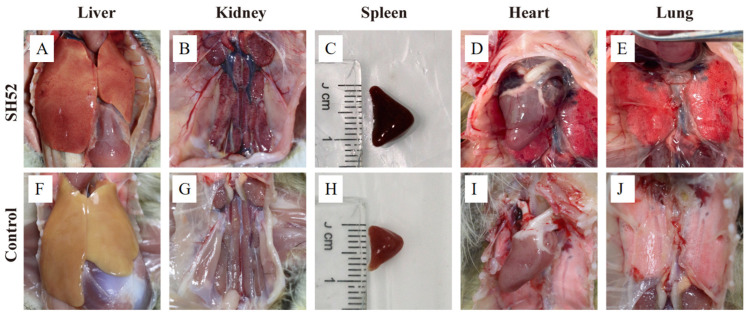
Histological changes in ducks infected with DHAV-3 SH52 strain. (**A**) Livers from challenged ducklings showed enlargement with multifocal subcapsular petechiae. (**B**) Kidneys displayed swelling and cortical hemorrhage with gray-white necrotic foci. (**C**) Spleens exhibited congestion presenting diffuse dark-red discoloration. (**D**) The heart was enlarged with pericardial dilation and patchy hemorrhages visible on the epicardium. (**E**) The lungs were congested and edematous. (**F**–**J**) Control specimens demonstrated absence of gross lesions.

Postmortem examination of deceased ducklings revealed characteristic gross pathological changes. Infected ducklings exhibited widespread hemorrhages across multiple internal organs. Notably, the liver was enlarged with distinct punctate hemorrhages on its surface. The kidneys were swollen and hemorrhagic, with grayish-white necrotic foci. The spleen was enlarged and hemorrhagic. The heart was enlarged with pericardial dilation, and patchy hemorrhages were visible on the epicardium. Additionally, the lungs were congested and edematous. In contrast, no significant gross lesions were observed in the organs of the PBS-infected control group (Figure 4).

**Figure 5:**
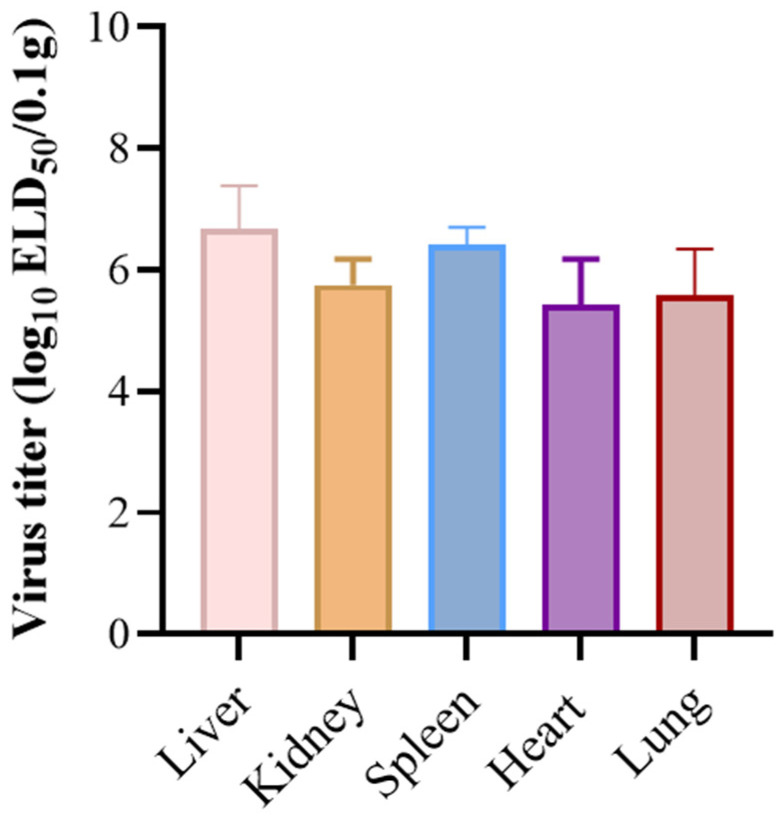
The virus titers of different tissues. SH52 replication was detectable in livers, kidneys, spleens, hearts, and lungs of ducks that died within 3 days post-challenge in the 10^6.5^ ELD_50_ group. Experimental data were presented as mean ± standard deviation. Error bars represent the standard deviation of the mean (*n* = 3 represents the virus titers of three tissue homogenates prepared from three different ducklings); tissue viral loads were analyzed by one-way analysis of variance (ANOVA) using GraphPad Prism 9.5 software.

To determine the viral titers in tissues, the supernatant from homogenized organs of deceased ducklings was diluted and inoculated into SPF duck embryos via the yolk sac route. The viral loads were analyzed by GraphPad Prism 9.5 software (Figure 5). The results showed that DHAV-3 SH52 replication was detectable in heart, liver, spleen, lung, and kidney tissues. The livers exhibited the highest viral titer, reaching 10^6.67^ ELD_50_/0.1 g, while the viral loads in the heart, spleen, lung, and kidney were in the range of 10^5.42^–10^6.42^ ELD_50_/0.1 g. These results indicated that DHAV-3 replicates extensively in various organs of shelducks.

**Figure 6:**
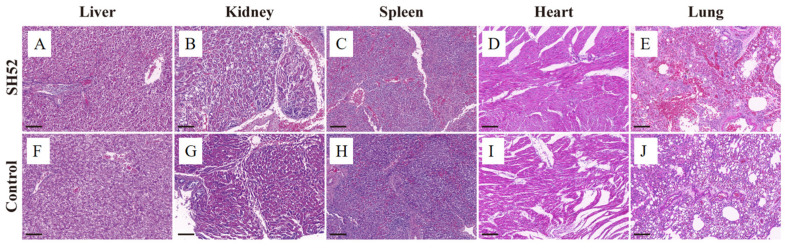
Pathological alterations in experimental tissues. (**A**) Liver degeneration and necrosis with cellular atrophy and cytolysis. Scale bar = 100 μm. (**B**) Kidney tubular epithelial degeneration and necrosis featuring nuclear pyknosis and minimal inflammatory infiltrate. (**C**) Disrupted periarteriolar lymphoid sheaths with lymphoid hyperplasia in spleen tissues. (**D**) No obvious pathological findings in the heart. (**E**) Interstitial hemorrhage in the lungs, proliferation of fibrous connective tissue, and inflammatory cell infiltration; (**F**–**J**) Absence of significant pathology in control specimens.

Histopathological examination of DHAV-3 SH52-infected ducklings revealed significant hepatic degeneration and necrosis. Liver tissues exhibited lobular disorganization with cellular atrophy and cytolysis, and abundant necrotic debris was present within the interstitium. The kidney sections showed marked tubular epithelial degeneration and necrosis, characterized by nuclear pyknosis, luminal structural effacement, interstitial fibrosis with fibroplasia, and minimal inflammatory cell infiltration. The spleen demonstrated dissolution of periarteriolar lymphoid sheaths, complete effacement of corticomedullary demarcation, and erythroid hyperplasia in the red pulp. Heart tissue appeared normal with no significant pathological changes observed. Lung tissue showed interstitial hemorrhage, along with degeneration and necrosis of the epithelial cells in the pulmonary capillary network. Fibrosis was noted in the necrotic areas, accompanied by proliferation of fibrous connective tissue and significant inflammatory cell infiltration (Figure 6).

Based on semi-quantitative scoring of gross lesions, DHAV-3 SH52 infection induced severe hepatic damage with mean scores of “+++”, while the kidneys and spleen exhibited moderate pathology with mean scores of “++”. The lungs and hearts demonstrated milder gross involvement with mean scores of “+” (Table 1). Histopathological evaluation further supported these findings, revealing severe lesions in the liver with mean scores of “+++”, substantial damage in both kidneys and lungs with mean scores of “++”, and moderate pathological changes in the spleen with mean scores of “++”. Notably, cardiac tissues showed no significant histopathological alterations (Table 2). These quantitative results indicate the liver may serve as the primary target organ, followed by substantial renal and pulmonary involvement, demonstrating the multi-organ tropism of DHAV-3 SH52 in ducklings.

## 4. Discussion

In this study, a DHAV-3 strain SH52 was isolated and identified. Phylogenetic analysis revealed that SH52 belongs to sub-clade 3-a, a distinct evolutionary clade of strains prevalent in China in recent years. And SH52 shared high homology with DHAV-3 strains reported in GenBank, particularly with the DHAV-3 A/dk/CHN/AH07/2018, exhibiting a similarity of up to 98.9%. This close genetic relationship suggests that the DHAV-3 SH52 strain is closely related to prevalent strains in China in recent years [23]. Notably, the branch containing SH52 also includes multiple strains reported in the literature as highly pathogenic. This phylogenetic clustering suggests that this lineage may share certain key genetic determinants leading to their common hypervirulent phenotype, which aligns with the 100% mortality rate observed in our animal experiments. We utilized the complete genome sequence for phylogenetic reconstruction, thereby avoiding the potential misclassification risks associated with relying on partial genes. The whole genome provides a stronger phylogenetic signal and higher resolution, enabling us to more reliably determine the evolutionary position of SH52, which is crucial for accurate molecular epidemiological tracing.

Animal infection experiments demonstrated that the DHAV-3 SH52 strain has high lethality in 8-day-old SPF shelducks. In groups inoculated with viral doses of 10^6.5^ ELD_50_ and 10^5.5^ ELD_50_, mortality reached 100% within 2 dpis, accompanied by severe gross and histological lesions in multiple organs, including the heart, liver, spleen, lung, and kidneys. The result is consistent with previous studies reporting that infection with virulent DHAV-3 strains causes high pathogenicity and mortality rates of up to 90–100% [24,25]. Additionally, the group inoculated with 10^4.5^ ELD_50_ and 10^3.5^ ELD_50_ exhibited a 60% and 50% mortality rate, respectively. The median lethal dose (LD_50_) of SH52 was calculated to be 10^−3.53^/0.1 mL, indicating the LD_50_ was approximately 10^3.5^ ELD_50_, providing a basis for determining the challenge dose in subsequent experiments. In practical applications, DHAV-3 vaccines are generally administered to 1-day-old ducks, followed by a challenge with the virulent strain 7 days later. To better evaluate the efficacy of the vaccine, we chose 8-day-old ducks for our infection experiments; the established challenge model in this study provided an ideal platform for evaluating whether the vaccine can offer sufficient protection when administered to 1-day-old shelducks and challenged 7 days later.

Viral load detection results were highly consistent with the site of pathological damage, particularly for the highest virus titer in the liver. This was closely related to the hepatophilic characteristics of DHAV-3 A/dk/CHN/AH07/2018 [8]. In the postmortem examination, the heart showed macroscopic changes such as enlargement and pericardial dilation, but histopathological examination revealed no significant microscopic lesions. This discrepancy may be due to indirect effects of systemic inflammation or individual variability in the response to viral infection; the minimal gross lesion score (+) for pericarditis, alongside the absence of histopathological alterations in the hearts, reinforces this interpretation. In addition, histological observations revealed extensive degeneration and necrosis of liver cells, necrosis of renal tubular epithelium, and destruction of splenic lymphoid structures, which are consistent with the typical pathological features of DHAV-3 infection reported in previous studies [26]. These findings further confirm the high pathogenicity of this strain.

Viral load assessment confirmed DHAV-3 replication in multiple organs. Although statistical significance was not achieved, the liver consistently demonstrated the highest mean viral titer, correlating with the most severe histopathological damage observed. Gross lesion scoring further supported these findings, with the liver showing the most severe pathology (+++), followed by moderate changes in the kidneys and spleens (++). Collectively, these virological and pathological results strongly suggest that the liver may serve as the primary target organ for the DHAV-3 SH52 strain isolated in this study. This feature is significant for vaccine development, and future antigen design could focus on inducing immune responses against liver-targeted infections to enhance protective efficacy. Given the liver’s central role in viral replication and pathology, it can serve as an indicator organ for evaluating vaccine-induced protection in challenge experiments. Monitoring viral load and pathological changes in the liver of surviving ducks can provide critical insights into the efficacy of vaccines against DHAV-3 infections.

Compared with mild to moderate pathogenic strains reported in other studies, SH52 has a more severe mortality rate and tissue damage, which may be related to specific genomic variations, such as amino acid substitutions in coding regions like VP1 or 3Dpol, which have been shown to be associated with increased virulence [27]. Future research should employ reverse genetic technology to functionally validate the key virulence determinants of SH52, thereby elucidating its pathogenic mechanisms.

In summary, a highly pathogenic DHAV-3 strain SH52 was successfully isolated and identified in this study. The phylogenetic analysis indicated that the DHAV-3 SH52 strain belongs to the currently circulating strains in China and an animal challenge model was established for evaluating the efficacy of vaccines in the future.

## Figures and Tables

**Table 1:** The gross lesions and score in DHAV-3 SH52-infected ducklings.

Lesion	DHAV-3 SH52	Controls
Hepatitis with Petechial hemorrhage	+++	-
Mottled kidneys with hemorrhage	++	-
Splenomegaly with Subcapsular hemorrhage	++	-
Pericarditis	+	-
Pneumonia	+	-

Score: “-” for no lesions, “+” for mild (5–25% involvement), “++” for moderate (26–50%), and “+++” for severe (>50%).

**Table 2:** The histopathological lesions and score in DHAV-3 SH52-infected ducklings.

Organ	Lesion	DHAV-3 SH52	Controls
Liver	Degeneration	+++	-
	Necrosis	+++	-
	Cellular atrophy	+++	-
	Cellular cytolysis	++	-
Kidney	Tubular epithelial degeneration	+++	-
	Necrosis	++	-
	Nuclear pyknosis	+++	-
	Inflammatory infiltrate	+	-
Spleen	Disrupted periarteriolar lymphoid	++	-
	Lymphoid hyperplasia	+	-
	Necrosis	-	
Heart	Degeneration	-	-
	Necrosis	-	
	Dilated interstitial blood vessels	-	-
Lung	Interstitial hemorrhage	++	-
	Necrosis	-	
	Proliferation of fibrous connective tissue	++	-
	Inflammatory cell infiltration	+++	-

Score: “-” for no lesions, “+” for mild (5–25% involvement), “++” for moderate (26–50%), and “+++” for severe (>50%).

## Data Availability

The original contributions presented in this study are included in the article/Supplementary Material. Further inquiries can be directed to the corresponding authors.

## Supplementary Materials

The following supporting information can be downloaded at https://www.mdpi.com/article/10.3390/microorganisms13122652/s1, Table S1. Primers used in this study.
