# Supplementary material for: Isolation and Characterization of a Duck Hepatitis A Virus Type 3 SH52 Strain in Ducklings
Source: Microorganisms. 2025 Nov 22;13(12):2652. doi: 10.3390/microorganisms13122652 (PMC12734566; doi:10.3390/microorganisms13122652)
Supplement: Supplementary file 1 [file microorganisms-13-02652-s001.zip › microorganisms-3954704-supplementary.pdf]

Table S1. Primers used in this study.

Primer Name	Sequence (5′→3′)	Target Gene	Size
DPV-REP-F	CAAACGGGGAGGGCAAAATAAGA	VP2	600 bp
DPV-REP-R	GTGGTCGCAGGTCCGTAGAGC		
DuCV-F	CGGCGCTTGTACTCCGTACTC	Rep	619 bp
DuCV-R	CCCGCGTGGTTTGTAATACTTG		
DAdV-3-F	GTACCGCCTTCGAGAACACA	Fiber1	1014 bp
DAdV-3-R	GCTGTCTGATTCTGGTGATC		
DHAV-3-F	GAAATCTGCACTCAATGGAGAG	VP1	286 bp
DHAV-3-R	CCCAGGAAATGATTGGTCAG		
NDRV-F	GCATGAACATGCCAGTTGAG	S1	300 bp
NDRV-R	AAGCCATAACGATGGCAGTC		
DTMUV-F	CATAGGCTGGAATCTGGGAAC	E	300 bp
DTMUV-R	TCTGGATTCTGTCGTCACGTC		
DERSV-F	CCAGCCTCGCAAGACTAAAT	3D	803 bp
DERSV-R	CAGAACACATACCTCCCTCAAC		
DHAV-1-F	CAGTTTACCGCCCCACTCTAT	VP1	699 bp
DHAV-1-R	TGGCTTCCACCTCCTCTTCAT		
Oligo dT-RA	TTTTTTTTTTTTTTTTTTCTGATCTAGACCTGCAGGCTCGAG	-	-
DHAV-3-P1-F	GTCTCATCATTTTGGCAAAGTTTGAAAGCGGC	-	2872 bp
DHAV-3-P1-R	AGGGAAAAAGATCTGCTAGCGAGCTCCATCTAGAAATTGAATCAGACCAATCTCGAAATGTC		
DHAV-3-P2P3-F	GTCTCATCATTTTGGCAAAGCATTTTGGCAAAGGAGCTCCATCTAGAAATTGAATCAGACCAATCTC	-	4985 bp
DHAV-3-P2P3-R	AGGGAAAAAGATCTGCTAGCCTGATCTAGACCTGCAGGCTCGAG		

The underlined sequences indicate the homologous arms. -, indicates that the item is not applicable.
